# Synovial effusion and synovial fluid biomarkers in psoriatic arthritis to assess intraarticular tumor necrosis factor-α blockade in the knee joint

**DOI:** 10.1186/ar3090

**Published:** 2010-07-19

**Authors:** Ugo Fiocco, Paolo Sfriso, Francesca Oliviero, Pascale Roux-Lombard, Elena Scagliori, Luisella Cozzi, Francesca Lunardi, Fiorella Calabrese, Maristella Vezzù, Serena Dainese, Beatrice Molena, Anna Scanu, Roberto Nardacchione, Leopoldo Rubaltelli, Jean Michel Dayer, Leonardo Punzi

**Affiliations:** 1 Department of Clinical and Experimental Medicine, University of Padova, Via Giustiniani 2, Padova, 35128, Italy; 2 Immunology and Allergy Division, Geneva University Hospitals and University of Geneva, Rue Gabrielle Perret-Gentil 4, Geneva, CH-1211, Switzerland; 3 Department of Diagnostic Sciences and Special Therapies, University of Padova, Via Giustiniani 2, Padova, 35128, Italy; 4 Department of Orthopedics, Leonardo Foundation, Abano Terme General Hospital, Piazza Cristoforo Colombo 1, Abano Terme (PD), 35031, Italy; 5 Faculty of Medicine, CMU 1, rue Michel-Servet, Geneva, CH-1211, Switzerland

## Abstract

**Introduction:**

The purpose of this study was theevaluation of synovial effusion (SE), synovial fluid (SF) and synovial tissue (ST) biomarkers in relation to disease activity indexes to assess the response to intraarticular (IA) tumor necrosis factor (TNF)-α blockers in psoriatic arthritis (PsA).

**Methods:**

Systemic and local disease activity indexes (disease activity score (DAS); the Ritchie articular index (mRAI), erythrocyte sedimentation rate (ESR) and C-reactive protein (CRP); Thompson articular (THOMP) and joint articular (KJAI)-Index ) and ST samples were assessed at baseline, throughout treatment, and during the follow-up in 14 patients affected with PsA who underwent IA injections (0.5 ml to 12.5 mg) in the knee joint of etanercept (E) or placebo (P) once every two weeks for a 10-week period. Total SF white blood cell (WBC) counts (WBC/μl) and SF cytokine/chemokine (CK/CCK) levels were measured before IA-E at baseline, after IA-E, and as long as there were adequate amounts of SF for knee aspiration (post). Characterization of synovial mononuclear cell infiltration and synovial vessels was carried out in 8 out of 14 knees by staining serial sections of synovial tissue biopsies for CD45, CD3, CD68, CD31 and CD105.

**Results:**

At baseline, CRP and/or ESR were significantly correlated with SF-CK (interleukin- (IL-)1β, IL-1Ra, IL-6, IL-8) and CCK (CCL3). Post-IA injections, there was a decrease in SE in the knees in which aspiration following IA-E injection was possible as well as a significant reduction in SF WBC/μl and in SF-CK (IL-1β, IL-1Ra, IL-6 and IL-22). Pre- and post-IA-E injections, there were significant correlations between ST markers and SF-CK (IL-1β with CD45; IL-1β and IL-6 with CD31) and between SF-CCK (CCL4 and CCL3 with CD3). At the end of the study, there was a significant reduction in disease activity indexes (CRP, DAS, RAI, THOMP, KJAI) as well as in the ST markers (CD45; CD3).

**Conclusions:**

Synovial effusion regression is a reliable indicator of the response to IA TNF-α blockers in PsA patients as it is confirmed by the correlation between SF biomarkers to disease activity and synovial tissue inflammation.

## Introduction

Actively inflamed joints in psoriatic arthritis (PsA) patients unresponsive to systemic treatments [1] show comparable levels of functional [2] and radiological disease progression [3] compared to those in rheumatoid arthritis (RA).

Prominent vascular alterations just beneath the lining cell layer, reduced layer lining thickness, and lower CD68 expression are distinctive features of PsA synovitis with respect to RA [4,5]. Tumor necrosis factor-alpha (TNF-α) plays an important role in the chronic inflammation found in PsA patients, and its increased expression together with that of other pro-inflammatory cytokines, including interferon-γ (IFN-γ), interleukin (IL) -12, IL-15, IL-17 and IL-18, and in particular, IL-6 and IL-1β, have been demonstrated in PsA synovium [6,7]. Disease-related cytokines in synovial tissue may also promote osteoclast formation resulting in bone erosion [8].

While the efficacy of TNF-α-blocking agents in reducing disease activity in PsA patients [9,10] has been demonstrated, their actual mechanisms of action are not completely understood [11-13]. Recent research has made it possible to identify new genetic factors [14,15] and immunopathological mechanisms common to psoriasis and psoriatic joint inflammation [16,17].

Genetic risk factors have implicated the interleukin (IL)-23 pathway and the induction and regulation of type 17 T-helper (TH-17) cells in the pathogenesis of psoriasis [18,19]. Secretion of cytokines, such as IL-22 and IL-17, could, moreover, induce keratinocyte proliferation and skin inflammation [19,20].

Biomarkers have been used as surrogate treatment endpoints in preliminary, short-term, proof-of-concept studies [21], but only limited data concerning biological biomarkers in psoriasis and psoriatic arthritis are available. It has been seen that histological findings are not correlated with clinical disease parameters [5]. The expressions of RANK ligand and osteoprotegerin (OPG) are similar in non-psoriatic spondyloarthropathy (SpA) as compared to PsA spondyloarthropathy [8], but neither are related to the degree of systemic or local inflammation, nor are they significantly modulated by effective response to TNF-α blockers [16,22]. The need, therefore, of reliable biomarkers to assess disease progression in PsA is clearly indisputable [21].

The aim of this longitudinal study was to investigate synovial effusion (SE), synovial fluid (SF) and synovial tissue (ST) biomarkers in relation to local and systemic disease activity biomarkers to assess the outcome of intra-articular (IA) TNF-α blockade therapy on gonarthritis in PsA patients [23-25].

## Materials and methods

IA-treatment was assessed by means of a single blind comparison between IA-etanercept (E) and IA-placebo (P), administered once every two weeks for a 10-week period in all those patients not needing to drop-out because of drug inefficacy, with a cross-over after the first IA injection. Those needing to drop-out were included in the open-label extension part of the study during which four IA-E injections were administered once every two weeks. Each 0.5 ml IA-injection (E: 12.5 mg, placebo: NaCl) was administered in individual knee joints after synovial fluid aspiration. The mean cumulative IA-E dosage for all of the patients was 50 mg for both the blind and open-label extension study. The study protocol (Etanercept/TNR-001:n.878P) was approved by the local ethics committee (Padova, 20 September 2004) and all patients signed consent statements after being informed about the intent and the methodology of the study [26].

PsA was defined as the presence of both psoriasis and inflammatory arthritis, regardless of their rheumatoid factor (RF) status. All 14 patients participating in the study fulfilled the CASPAR (CLASsification criteria for Psoriatic ARthritis) classification criteria for PsA [27]. The psoriasis area and severity index (PASI) was less than 10 in these patients. Affected with active gonarthritis, which was characterized by pain, tenderness, and effusion, all of the patients were being treated with stable DMARD, steroid, and/or E systemic therapy.

### Assessment

Patients' responses to therapy were blindly assessed by the same investigator (LC).

SF cell counts (C/μl) were performed on all of the samples aspirated before IA-E injection throughout the entire study.

The primary efficacy endpoint utilized was the knee Thompson Articular Index (THOMP) [28], a sum of scores for each knee joint concerning pain on movement (0 to 3), soft tissue swelling (0 to 3) and warmth (0 to 3); (range 0 to 9). The secondary efficacy endpoint was: the Knee Joint Articular Index (KJAI), already validated in RA, PsA, and SpA-Knee Joint Synovitis (KJS) patients, likewise a sum of scores (0 to 14) for tenderness (0 to 3), joint swelling (0 to 3), the ballottement of patella or the *bulge sign *(0 to 2), the range of knee joint flexion (0 to 3) and extension (0 to 3) [29].

The systemic secondary endpoints were: (i) The modified Ritchie Articular Index (mRAI), a sum of scores assessing 30 joints for tenderness (0 to 3) including hand and foot Distal Interphalangeal Joints (DIP) with each side considered as a group score (DIP involvement is often observed in oligo-and poli-PsA patients) [30] and the Disease Activity Score (DAS). (ii) Erythrocyte Sedimentation Rate (ESR) (≤ 28 mm/h) and serum C-reactive Protein (CRP) values (≤ 0.5 mg/dl) were assessed at baseline and at the end of the study.

### Synovial fluid biomarkers

SF samples, aspirated from knee joints before the first IA-E injection, at baseline, and before each IA-E injection, were collected and frozen at -80°.

The *post-treatment *synovial effusion was defined as the last SF sample available for aspiration after one or more IA-E injections in each knee. The last SF sample available from each knee (post-treatment) underwent synovial effusion analysis. The SF samples were analysed for total white blood cell (WBC) counts (WBC/μl). The cytokines: IL-1β, IL-1 receptor antagonist (IL-1Ra), IL-6, IL-17, TNF-α, IFN-γ, the CXC chemokine IL-8, the CC chemokines, CCL2, the monocyte chemoattractant protein-1 (MCP-1), CCL3, the macrophage inflammatory protein (MIP-1α) and CCL4, the macrophage inflammatory protein-1β (MIP-1β), were measured using a commercially available multiplex bead immunoassay, based on the Luminex platform (Fluorokine MAP Multiplex Human Cytokine Panel A, R&D Systems, Minneapolis, MN, USA) following the manufacturer's instructions. Normal serum values were those established in 50 healthy blood donors. IL-22 was measured using a commercially available ELISA kit (Quantikine Human IL-22, R&D Systems). SF was centrifuged before determinations were made at 1,000 g to remove cells and debris.

### Synovial biopsy

Synovial biopsies were carried out during arthroscopy while patients were under the effect of anaesthesia, no earlier than two weeks before the first IA-E injection and no later than two to four weeks after the last one. Synovial specimens were obtained targeting the areas of intense synovial hyperemic proliferation. Multiple biopsy samples from each patient were stored in paraformaldehyde and embedded *en bloc *in paraffin.

### Immunohistochemistry

Characterization of synovial mononuclear cell infiltration and synovial vessels was carried out in consecutive serial sections of synovial biopsies obtained from eight patients before the first and after the last IA-E injection. In particular they were immunostained by using the following antibodies: CD3 (Novocastra, Newcastle Upon Tyne, UK), CD68, CD45 (clone 2B11), CD31 (clone JC70A), CD105 (SN6h) (Dako Cytomation, Glostrup, Denmark). All the parameters were measured by computer-assisted morphometric analysis (Image Pro-plus version 5) and a 2 mm square area was evaluated.

### Statistical analysis

Mean and standard deviations were used as descriptive statistics. Changes over time of selected outcomes and biological markers after IA-E treatment were evaluated using the non-parametric Wilcoxon Signed Rank test. All analyses were performed using SPSS software (SPSS 15.0 (SPSS Inc., Chicago, IL, USA). The Spearman Rank test was used for correlation analysis.

## Results

The main clinical details as well as systemic disease activity indexes of all patients are listed in Table 1. Four PsA patients were being treated with parenteral E from the time of screening to the end of the IA treatment period.

**Table 1:** Clinical, demographic characteristics and systemic disease activity indexes of psoriatic arthritis patients

Knee	Disease duration (years)	Gonarthritis duration (years)	Treatment at study entry	ESR		CRP		DAS		mRAI	
							
				PRE	POST	PRE	POST	PRE	POST	PRE	POST
**1**	10.3	10.3	MTX; PN	46.4	88.4	1.78	0.33	3.21	3.42	8	8
**2**	8.4	6.4	PN;MTX;CSA;E	40.3	29.0	2.77	0.72	3.44	3.33	12	12
**3**	12.5	10.5	PN	23.2	22.2	0.53	0.02	2.33	1.25	3	0
**4**	8.6	8.6	LEF;E	72.5	33.8	0.56	0.56	2.78	2.59	4	4
**5**	22	5.7	PN	12.0	13.0	0.85	0.53	2.88	2.47	6	5
**6**	11	11	MTX	15.6	22.7	0.10	0.00	2.69	3.00	7	9
**7**	9	9	SSZ	15.0	11.0	0.66	0.39	3.18	2.42	12	5
**8**	6.5	4.3	MTX; PN; E	6.0	9.9	0.32	0.80	2.65	1.87	10	2
**9**	4	3.5	MTX; SSZ	18.1	3.6	2.93	0.24	2.52	0.71	5	0
**10**	9	15	CSA;PN; E	30.0	20.0	0.00	0.00	3.01	2.40	2	1
**11**	4.8	4.5	MTX;CSA	29.4	25.2	0.08	0.08	2.48	1.89	4	1
**12**	2.5	2.5	MTX; PN	14.3	6.8	0.70	0.20	3.97	3.40	20	18
**13**	8.5	2	SSZ; MTX	41.2	27.2	0.48	0.14	2.77	1.98	3	1
**14**	5.5	3.5	SSZ	13.6	2.2	0.26	0.26	3.22	0.75	9	0

CRP, C-reactive protein (≤ 0.5 mg/dl); CSA, cyclosporin; DAS, disease activity score; E, etanercept; ESR, erythrocyte sedimentation rate (≤ 28 mm/h); HCQ, hydroxychloroquine; LEF, lefluonomide; mRAI, modified Ritchie articolar index; MTX, methotrexate; PN, prednisone; PsA, psoriatic arthritis; Pt, patient; SSZ, sulphasalazine.

**Figure 1:**
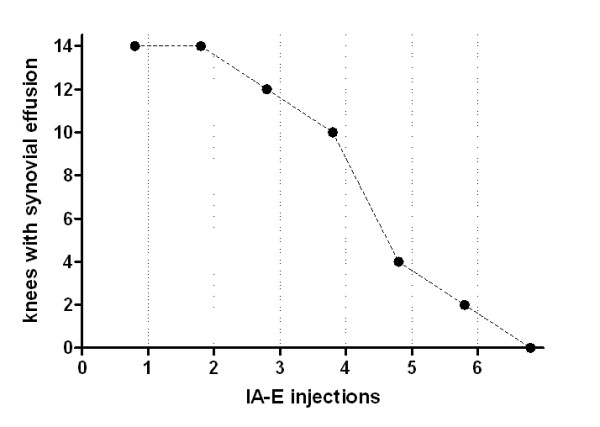
**Follow-up of synovial fluid effusion during IA-E treatment**.

**Figure 2:**
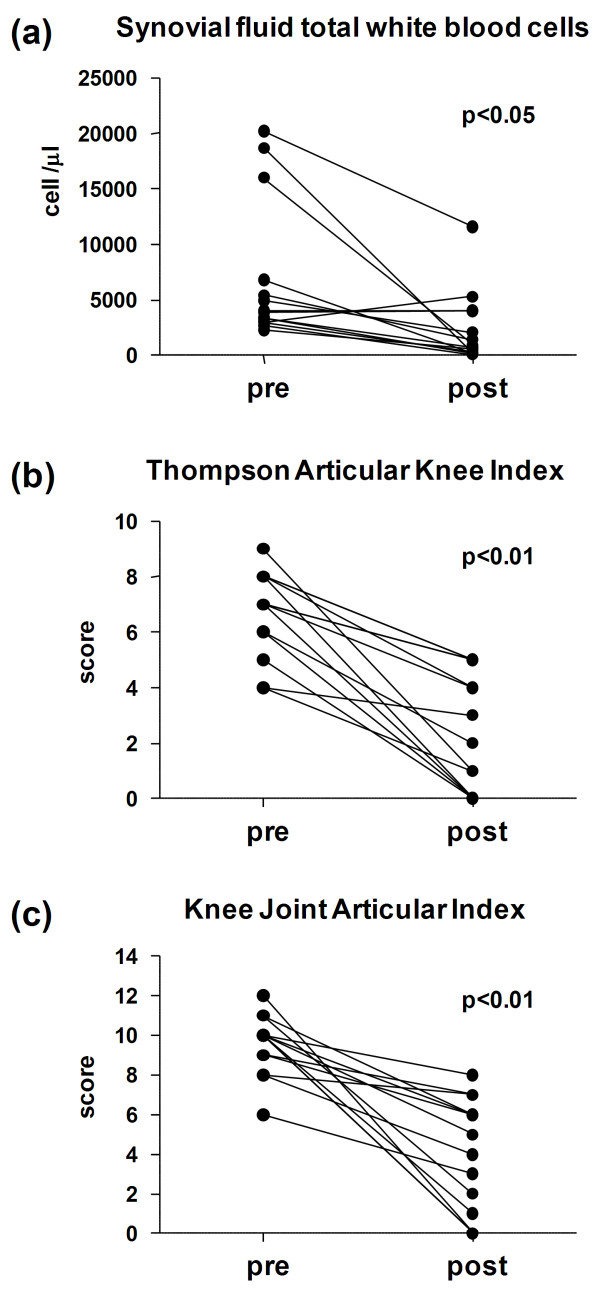
**SF total white blood cells and knee clinical indexes before and after intra-articular TNF-α blockade**. Synovial fluid total white blood cells **(a) **Thompson Articular Knee Index **(b) **and Knee Joint Articular Index **(c) **in 14 PsA patients before and after intra-articular TNF-α blockade: pre, at baseline; post, at the time the last synovial fluid sample was available for aspiration after one or more IA-E injections in each knee. Significance by Wilcoxon rank test.

SF samples were aspirated immediately before the first IA injection from all 14 knees. There was a decrease in SE in the knees in which aspiration following IA-E injection was possible. In 10 knees the effusion disappeared before the fifth IA-E injection (Figure 1). There was a statistically significant reduction in synovial fluid WBC/μl when pre and post IA-E values were compared. (Figure 2a).

The Thompson Articular Knee Index values were significantly reduced (Figure 2b) in the 14 knees at the *post-treatment *assessment (at the time the last SF sample was available) as well as at the end of the study (two weeks after the last IA-E injection), and there were no differences in the results between these two points in time.

A statistically significant reduction in the systemic biological (CRP: 0.86 ± 0.95 and 031 ± 0.26, *P *= 0.019) and clinical (DAS: 2.93 ± 0.43 and 2.24 ± 0.90, *P *= 0.002, mRAI: 7.50 ± 4.88 and 4.71 ± 5.37, *P *= 0.011) disease activity indexes was observed at the end of the study, as well as statistically significant reduction of local composite (THOMP; KJAI) disease activity indexes at the *post-treatment *assessment (Figure 2b, c) and the end of the study.

Pre-treatment IFN-γ was undetected in all the SF samples at baseline and throughout the study. Several cytokines/chemokines (IL-1β, IL-1Ra, IL-6, IL-8, MCP-1/CCL2, MIP-1α/CCL3 and MIP-1β/CCL4 as well as IL-17 and IL-22) were detected.

There were significant correlations in some pre-treatment systemic and biological disease activity indexes and specifically between CRP and IL-1β, IL-1Ra, IL-6 SF levels as well as between ESR and IL-1β, IL-1Ra, IL-8 and MIP-1α/CCL3 SF levels (Table 2). There were, moreover, significant correlations in the IL-1β, IL-6, IL-1Ra SF biomarkers, which were correlated to one another. There was also a correlation between IL-22 and TNF-α. Both IL-8 and IL-6 were correlated to MIP-1α/CCL3 and MIP-1β/CCL4, respectively. Finally, MCP-1/CCL2, IL-1Ra and MIP-1α/CCL3 were correlated to one another.

**Table 2:** Correlations between synovial fluid biomarker levels and biological disease activity indexes at baseline in 14 knees

Spearman's rank correlation coefficients		
	**CRP**	**ESR**

**IL-1β**	0.61*	0.57*
**IL-1Ra**	0.57*	0.54*
**IL-6**	0.69**	ns
**IL-8**	ns	0.54*
**MIP1-α**	ns	0.67**
**TNF-α**	ns	ns
**IL-17**	ns	ns
**IL-22**	ns	ns

Significance by Sperman's rank test: * *P *< 0.05; ** *P *< 0.01; ns, not significant.

**Figure 3:**
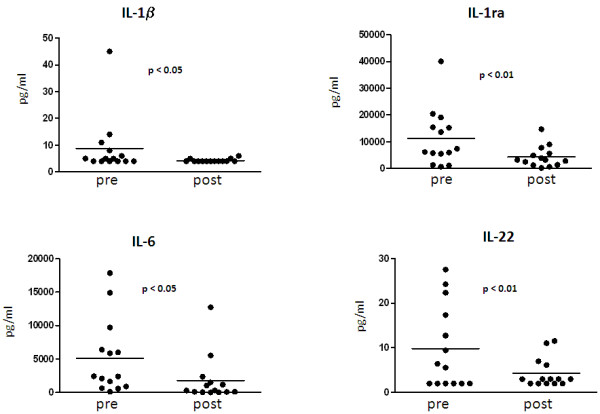
**Synovial fluid cytokines levels in 14 PsA knees before and after intra-articular TNF-α blockade**. pre, at baseline; post, at the last SF sample available for aspiration after IA-E injections. Significance by Wilcoxon rank test.

There was a statistically significant reduction in post-treatment IL-1β, IL-1Ra, IL-6 and IL-22 levels with respect to basal values (Figure 3).

A significant correlation was observed at baseline between IL-1β and CD45. Both IL-1β and IL-6 were correlated with CD31. There was a correlation between MIP-1β/CCL4 and CD3-ST pre-injection values and between MIP-1α/CCL3 and CD3-ST post-injection levels. TNF-α blockers induced a significant down-regulation in CD45 (1157.0 ± 712.9 and 545.8 ± 253.2, *P *= 0.007) and CD3 (402.8 ± 203.0 and 224.8 ± 107.7, *P *= 0.039) ST expression.

## Discussion

The aim of this longitudinal study was to evaluate SE, SF and ST biomarkers to assess the response to intra-articular TNF-α blockade therapy in PsA patients. The study's most striking finding was that synovial effusion disappeared in the knees of PsA patients, indicating that the therapy was effective. Its regression in the knees with enough SE to permit aspiration and analysis, the significant reduction in synovial fluid WBC counts as well as in the SF-CK (TNF-α, IL-1β, IL-1Ra, IL-6 and IL-22) indicate that IA-E injections have a local effect on synovial inflammation.

The delay in the response of several knees of PsA patients after IA TNF-α blockade, may indicate that drug dosage was insufficient to control local knee disease activity.

The expression of proinflammatory cytokine/chemokine at baseline in the SF of the PsA patients is consistent with previous findings in the SF [31,32] and in the ST [5-7,11,33] of these patients. IL-6 concentrations were similar in the SF of RA and SpA to PsA [34] while higher IL-17 levels were observed in the SF of SpA to PsA [35-37]. At baseline, IL-1β was correlated to IL-6 levels in SF as well as to CD45 expression in ST and MIP-1 β/CCL4 was correlated to post CD3 expression in ST.

High circulating [38] and SF levels of (MCP-1)/CCL2 have been observed in RA, PsA and SpA patients [34,39,40] and were also associated to the response to etanercept in RA patients [41]. In PsA, localized CCL2 production was correlated to the T cell infiltration of PsA synovium [42]. In accordance with previous reports, our findings in the SF of PsA patients support the hypothesis that (MCP-1)/CCL2, MIP-1α/CCL3 and MIP-1β/CCL4 chemokines play an important role in PsA development [43].

Elevated IL-22 expression in the SF of PsA patients, a novel finding in our patients, suggests that the Th17 system may have an underlying role in both skin [20] and joint involvement. The potential proinflammatory function in joints of IL-22, a cytokine of the IL-10 family, has been suggested by IL-22 mRNA expression by macrophages and fibroblasts, by MCP-1/CCL2 production and fibroblast proliferation of RA patients [44] and by the promotion of osteoclastogenesis in collagen induced arthritis [45].

Alterations in CD45 and CD3 ST expressions are in agreement with the decrease in the global cellular infiltration and T-lymphocytes, already found to be associated with active systemic anti TNF-α treatment in both Ra and PsA [46-48].

With regard to serum biological biomarkers, IL-6, IL-ra, IL-10 and ESR have been studied in PsA, but only IL-1ra and ESR have been found to reflect disease activity [49,50]. ESR and C-reactive protein were found to be closely correlated to TNF-α blockade response [51,52], but not to cytokine levels [21].

No previous study has evaluated the correlation between SF biomarkers and systemic and local composite disease activity indexes in PsA patients [53,54]. Synovial fluid analysis carried out in our patients during this study indicates that SF biomarkers are correlated to ST inflammation markers and to local and systemic indexes of disease activity in PsA.

Serum IL-17 does not seem to be influenced by TNF-α blockade following etanercept and infliximab both in SpA and in RA [35,55]. According to experimental data, TNF-α may lead to an increased activity of other proinflammatory pathways [56-58]. The fact that IL-22 and IL-17 do not react in the same way in the SF of PsA would seem to indicate that they have distinct regulatory pathways [59,37] and different cellular sources [60-62].

This study has important limitations: use of monovariate statistical methods, the limited number of patients studied, the concomitant DMARD treatment, the differences in the drug doses utilized, and the number of injections administered. Larger, controlled studies are, therefore, clearly warranted to further assess their clinical relevance.

## Conclusions

Regression of synovial effusion is a reliable indicator of the response to intra-articular TNF-α blockade therapy in PsA patients as it is confirmed by the correlation of SF biomarkers to disease activity and synovial tissue inflammation.

## Abbreviations

CASPAR: CLASsification criteria for Psoriatic ARthritis; CK: cytokine; CCK: chemokine; CRP: C-reactive protein; DAS: Disease Activity Score; DIP: Distal Interphalangeal Joints; E: etanercept; ESR: erythrocyte sedimentation rate; IA: intra-articular; IFN-γ: interferon-γ; IL: interleukin; IL-1Ra: IL-1 receptor antagonist; KJAI: Knee Joint Articular Index; KJS: knee joint synovitis; MCP-1: monocyte chemoattractant protein-1; MIP-1α: macrophage inflammatory protein; MIP-1β: macrophage inflammatory protein-1β; mRAI: modified Ritchie Articular Index; OPG: osteoprotegerin; P: placebo; PASI: psoriasis area and severity index; PsA: psoriatic arthritis; RA: rheumatoid arthritis; RF: rheumatoid factor; SE: synovial effusion; SF: synovial fluid; SpA: spondyloarthropathy; ST: synovial tissue; TH-17: type 17 T-helper; THOMP: Thompson Articular Index; TNF-α: tumor necrosis factor alpha; WBC: white blood cell.

## Competing interests

UF has received speaking fees and/or research grants from Wyeth Lederle, Schering Plough and Bristol-Myers Squibb. LP has received speaking fees and/or research grants from Wyeth Lederle, Schering Plough, Bristol-Myers Squibb, Abbott International, Rottapharm, Fidia Farmaceutici and Roche. The authors declare that they have no other competing interests.

## Authors' contributions

UF was responsible for the study concept and design, analysis and interpretation, and drafting the manuscript. PS participated in the design of the study and performed the statistical analysis. FO performed the statistical analysis and helped to draft the manuscript. PRL carried out the immunoassays. ES participated in the assessment of the patients. LC assessed the patients' response to therapy. FC was involved in the pathological diagnosis and FL in immunohistochemical characterization. MV participated in the design of the study and in the assessment of the patients. SD participated in the assessment of the patients. BM helped to carry out the immunohistochemistry. AS helped to carry out the immunoassays. RN carried out the arthroscopy and synovial biopsies. LR performed the diagnostic imaging. JMD participated in the design of the study and revised the manuscript. LP was responsible for the study concept and revising the manuscript. All authors read and approved the final manuscript.
